# Impact of Channel Effects on Radiation-Hardened InAlGaN HEMTs for Low-Earth-Orbit Applications

**DOI:** 10.1021/acsomega.5c02838

**Published:** 2025-05-21

**Authors:** Shao-Kuan Lee, You-Chen Weng, Chien-Yuan Huang, Edward-Yi Chang, Yuan-Chieh Tseng

**Affiliations:** † International College of Semiconductor Technology, 34914 National Yang Ming Chiao Tung University, 30010 Hsinchu, Taiwan; ‡ College of Photonics, National Yang Ming Chiao Tung University, 71150 Tainan, Taiwan; § Department of Materials Science and Engineering, National Yang Ming Chiao Tung University, 30010 Hsinchu, Taiwan

## Abstract

This study investigates the impact of channel thickness effects on the radiation hardness of InAlGaN HEMTs under 90 MeV proton irradiation for low-earth-orbit (LEO) applications. Devices with varying channel thicknesses (50, 100, and 150 nm) were exposed to proton fluences ranging from 2 × 10^10^ to 2 × 10^13^ protons/cm^2^. Results show that the 100 nm channel thickness exhibits superior radiation hardness, maintaining higher mobility, lower sheet resistance, and superior DC and RF performance compared to other thicknesses, even at high proton fluences. Ionizing energy loss is identified as the dominant contributor to degradation, although both ionization and displacement damage mechanisms are observed. Gate leakage current remains relatively stable across all proton fluences and thicknesses due to the counteracting effects of irradiation-induced knock-on atoms. These findings highlight the importance of channel thickness optimization for enhancing the radiation tolerance of InAlGaN HEMTs in demanding applications.

## Graphical abstract

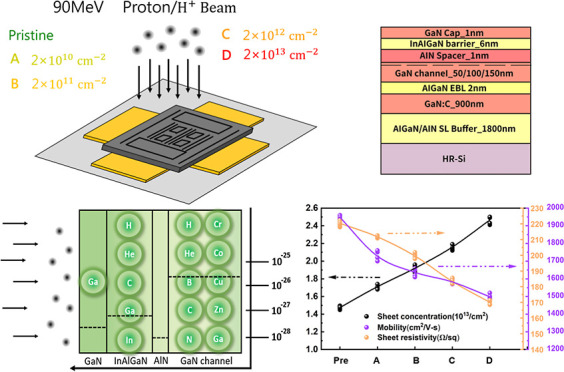

## Introduction

The growing demand for radiation-hardened high-speed electronics in space has intensified interest in GaN high-electron-mobility transistors (HEMTs).^1–9^ GaN’s superior radiation tolerance, attributed to its ability to accommodate lattice disorder, improves key parameters such as current density, linearity, and off-state leakage current.^4–6,10–14^ However, long-term exposure to high-radiation environments leads to performance degradation, including reduced saturation current, decreased transconductance, and increased leakage current.

To maximize GaN’s potential in space, understanding radiation effects is essential, particularly in addressing displacement damage (DD), total ionizing dose (TID), and single-event effects. Research has focused on defect mechanisms, impurity dehydrogenation, and reliability modeling.^4,5,15–19^ In this context, InAlGaN has emerged as a promising alternative to AlGaN and InAlN, offering higher spontaneous polarization for improved 2DEG confinement and charge density.^20,21^ However, deep-level traps in the buffer layer can degrade 2DEG performance, necessitating optimization with a high-mobility unintentionally doped GaN layer.^22,23^

Radiation-induced defect formation in epitaxial layers impacts device performance, making preirradiation layer design critical. GaN-on-silicon substrates, while attractive for high-frequency applications, face challenges such as AlN/Si interface losses and thermal management issues.^24–26^ Solutions include AlGaN/AlN superlattice buffers, which enhance crystal quality and enable thicker GaN growth, improving breakdown voltage and leakage current control.^27,28^ Carbon-doped GaN (GaN/C) back-barrier layers further enhance breakdown voltage but at the cost of reduced channel conductivity, highlighting a key design trade-off.^29,30^

Channel thickness also plays a crucial role in device performance. Thinner channels enhance RF performance and mitigate short-channel effects but increase trapping and thermal challenges, whereas thicker channels improve large-signal performance and reliability but degrade RF characteristics. Optimizing this trade-off is essential for space applications.^31–36^

Radiation testing is critical for ensuring device reliability in space, where exposure to electrons, protons, neutrons, and γ rays varies by orbital altitude. In low earth orbit (LEO), where reduced signal delay benefits systems like SpaceX Starlink, prolonged exposure necessitates rigorous qualification. Proton irradiation at ∼100 MeV with fluences of ∼10^11^ protons/cm^2^ simulates a typical five-year mission.^37–39^ This study examines the radiation response of InAlGaN HEMTs with varying channel thicknesses under 90 MeV proton fluences (10^10^ – 10^13^ protons/cm^2^) to identify the optimal design for LEO applications. By refining radiation-induced degradation models and optimizing key design parameters, InAlGaN HEMTs can enhance the reliability of high-speed electronics in space.

## Experimental Section

The InAlGaN HEMTs structure featuring the GaN/C doping/electron blocking layer (EBL) buffer was grown using the Veeco Propel Metal Organic Vapor Deposition (MOCVD) system on a high-resistivity 6-in. Si(111) substrate. The epitaxial layer structure of the device, from bottom to top, comprised an 1800 nm AlGaN/AlN superlattice (SL) buffer layer, a 900 nm GaN/C doping layer, a 2 nm Al_0.05 Ga_0.95N electron blocking layer, with three different thicknesses (50/100/150 nm) of unintentionally doped (UID) GaN channel layers, a 1 nm AlN spacer, a 6 nm I n_0.04_ A l_0.63_ G a_0.33_ N barrier layer, and a 1 nm GaN cap layer. The fabrication process of the HEMT device commenced with the formation of ohmic contacts, followed by achieving device isolation through multiple energy N^+^ implantations. A 200 nm gate was then created using electron beam lithography and dry etching through the PECVD-SiN_*x*_ layer, resulting in a slight recess of the GaN cap layer (∼1 nm). Subsequently, a second lithography step was carried out for Schottky gate fabrication, and interconnecting formation for multifinger source electrodes was established. These electrodes were connected with air-bridges formed by sputtered seed metal Ti/Au and thick Au electroplating. The source-drain distance (L_SD_) and gate width of the devices were set at 2 and 50 μm, respectively. Experimental Configuration for Radiation Studies utilized a 90 MeV proton beam with a 6 cm × 6 cm irradiation area to ensure uniform energy deposition across all samples. Four groups (A–D) of samples were exposed to varying proton fluences, ranging from 2 × 10^10^ cm^–2^ to 2 × 10^13^ cm^–2^, with each group differing by 1 order of magnitude, as depicted in Figure 1.

**Figure 1:**
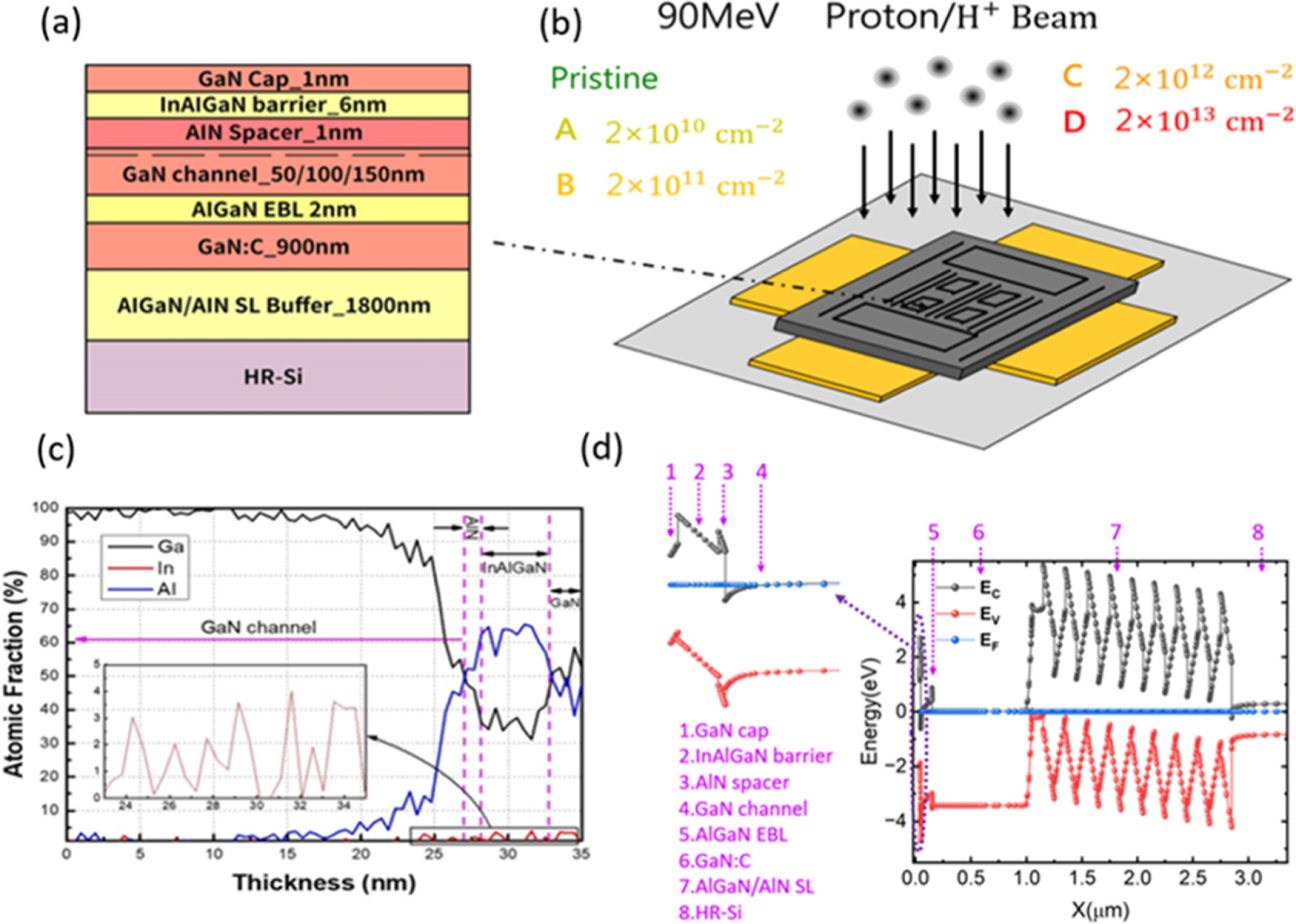
(a) Schematic cross-section of the InAlGaN/GaN-based epitaxial structure. (b) Illustration of the proton irradiation experimental setup; samples were irradiated at a fixed energy of 90 MeV with four different fluences, along with a pristine control sample (2 × 10^10^, 2 × 10^11^, 2 × 10^12^, and 2 × 10^13^ cm^–2^ correspond to groups A, B, C, and D, respectively). (c) TEM-EDS line scans of the epitaxial cross-section. (d) Simulated band diagram of the complete structure. The dashed arrows in the figure indicate the corresponding material structures, while the black, red, and blue lines represent the energy position distributions of the conduction band, valence band, and Fermi level, respectively. The position of the 2DEG is located between structure 3 and structure 4 (between the AlN spacer and the GaN channel).

To validate the structure, high-resolution transmission electron microscopy (TEM) and energy dispersive spectroscopy (EDS) scans were conducted on a cross-section of the epitaxy. The line scan of the GaN/InAlGaN/AlN/GaN interface in Figure 1b revealed the presence of In, Al, and Ga atoms within the InAlGaN barrier layer, with atomic percentages of approximately 4%, 63%, and 33%, respectively. Figure 1c displays Sentaurus TCAD (version 2023.9) simulations of the heterostructure band diagram, assuming identical top layers. The InAlGaN layer was modeled with a 4.8 eV bandgap and a total polarization charge of −8.54 × 10^–6^ C/cm^2^, and an electron affinity of 1.3 eV. These parameters were selected to represent optimal channel confinement. The incorporation of an EBL effectively restricts the two-dimensional electron gas (2DEG) density tail penetration into the underlying buffer layer. A reduction in channel thickness resulted in both a decreased peak 2DEG density and a slight reduction in the overall 2DEG sheet density (*n*_s_). Numerical integration across the simulated depth yielded *n*_s_ values of 0.9, 1.45, and 1.66 × 10^13^ cm^–2^ for 50, 100, and 150 nm channel thicknesses, respectively.

All epitaxial layers and devices were processed and characterized under identical environmental conditions (see Figure S1).

## Results and Discussion

Hall measurements for Preirradiation measurements were performed on four Van der Pauw structures patterned on each heterostructure­(seeTable S1). Sixteen 1 cm^2^ 100 nm channel thickness samples were prepared from a pristine epitaxial structure for testing. After all samples were measured, the 16 samples were divided into four groups, each corresponding to a subsequent irradiation fluence experiment (groups A, B, C, and D corresponded to proton irradiation fluences of 2 × 10^10^, 2 × 10^11^, 2 × 10^12^, and 2 × 10^13^, respectively). Each fluence group comprised four samples, and the values of testing for InAlGaN epitaxy structures at various fluences at room temperature are shown in Figure 2.

**Figure 2:**
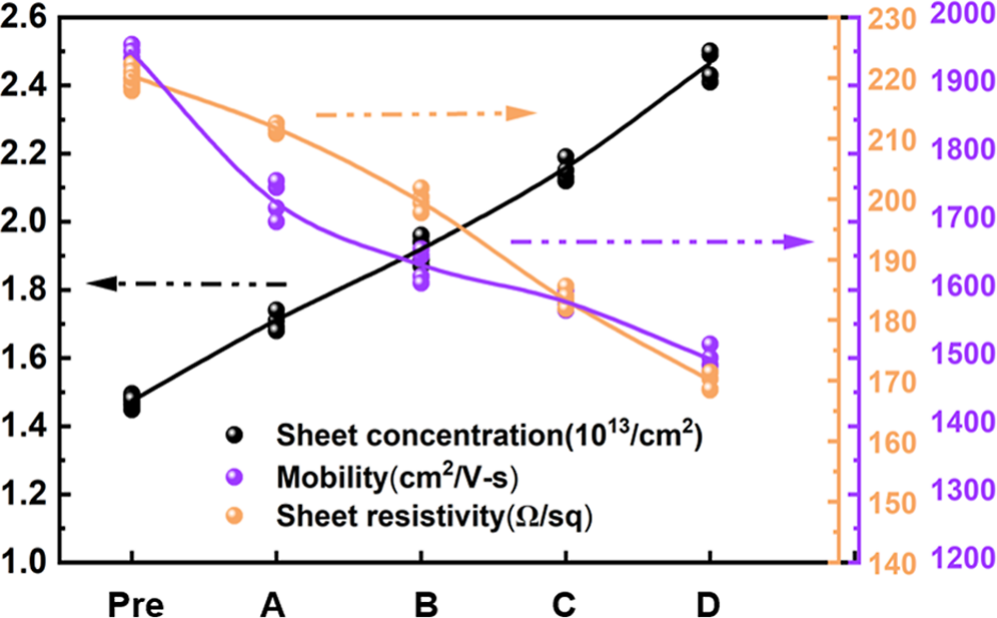
Influence of proton irradiation on sheet resistance, 2DEG mobility (μ), and carrier concentration­(*t*_ch_ = 100 nm). In the figure, “Pre” represents the measured data obtained before irradiation; A, B, C, and D represent the measured data obtained after the sample underwent proton irradiation with fluences of 2 × 10^10^, 2 × 10^11^, 2 × 10^12^, and 2 × 10^13^, respectively. The arrows corresponding to different colors indicate variations in scale, and the connected lines represent the average values derived from the points of different measurement results.

With increasing proton fluence, both mobility and sheet resistance (*R*_sh_) exhibited a consistent downward trend, following a predictable order-of-magnitude pattern. This decrease in mobility is primarily attributed to the introduction of radiation-induced defects (e.g., N, Ga, H, He vacancies, and interstitial atoms) created by direct proton impact.^43^ However, the 90 MeV protons also induce nuclear reactions within the GaN lattice, generating a cascade of secondary particles. These secondary particles further contribute to lattice damage by depositing their energy and creating additional defects throughout the material. Furthermore, this secondary particle cascade significantly amplifies the overall damage beyond that directly caused by the primary proton beam. Further analysis of the damage induced by postirradiation particles is provided in Figure 3.

**Figure 3:**
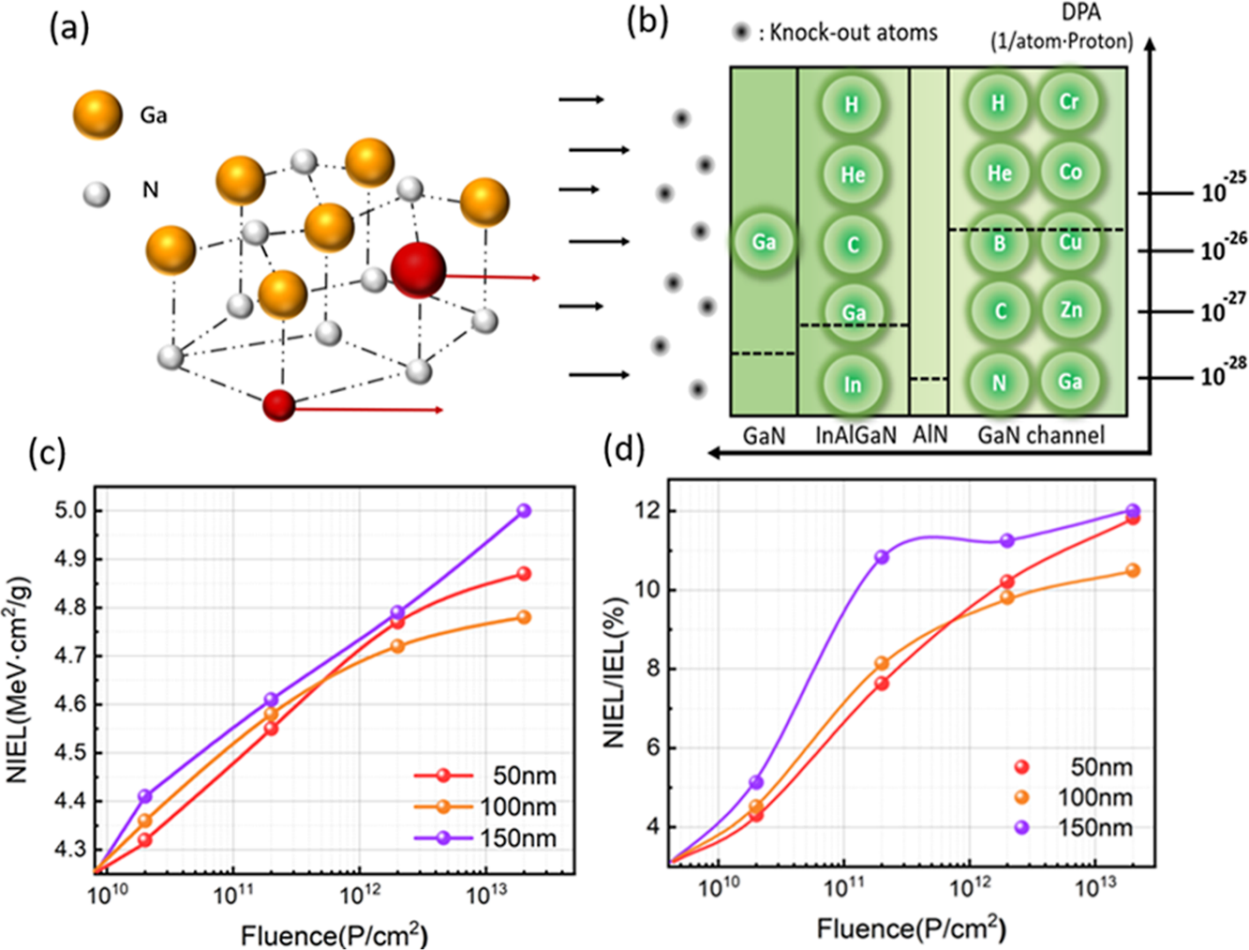
(a) Schematic of atomic displacement in wurtzite GaN following primary knock-on atom (PKA) impact; (b) the distribution of displaced atoms and displacement per atom (DPA) values across different layers; (c) nonionizing energy loss (NIEL) in samples of varying channel thickness under different proton fluences; and (d) the proportion of NIEL in the device as a function of fluence.

Conversely, carrier concentration (*n*_s_) steadily increased with fluence. This increase is because these radiation-induced defects, both from direct proton impact and secondary particle interactions, introduce additional charge carriers into the GaN lattice. The defects can create energy levels within the band gap, which can trap electrons, leading to an increase in the overall carrier concentration. Despite these changes, the material maintained its conductive properties without undergoing a type conversion (e.g., from n-type to p-type GaN). Electron conduction remained the dominant mechanism. Consequently, the observed decrease in *R*_sh_, coupled with the increase in *n*_s_ and decrease in mobility (μ), resulted in a material state resembling that of undoped GaN with a high defect concentration at relatively high fluences (in this experiment, fluence = 10^13^ p/cm^2^). The persistence of n-type conduction, even at high fluences, suggests that the density of donor-like defects introduced by the irradiation (both primary and secondary particle effects) exceeds the density of acceptor-like defects.

We also investigated the types and spatial distribution of atoms displaced from their lattice sites following irradiation. Figure 3a schematically illustrates the atomic displacement within a wurtzite GaN structure (used as a representative example) after imparting initial kinetic energy to a primary knock-on atom (PKA). Particle and Heavy Ion Transport code System (PHITS) simulations were then employed to determine the secondary ion yields and displacement per atom (DPA) values, providing further insight into the radiation damage. Figure 3b presents the elemental composition of the displaced atoms and the corresponding DPA values for each layer. In the GaN channel layer, the displaced atoms include H, He, B, C, N, Ga, etc., with DPA values ranging from 10^–25^ to 10^–28^. The GaN channel layer exhibits the highest DPA, indicating significant damage in the high-carrier-density 2DEG region due to proton irradiation, leading to degradation of device characteristics. Figure 3c shows that the nonionizing energy loss (NIEL) increases linearly with fluence for the 150 nm sample, while the increase is less pronounced for the 100 and 50 nm samples. The ionizing energy loss (IEL) remains approximately 1 order of magnitude higher than the NIEL across all thicknesses (Figure 3d), indicating that IEL dominates the damage mechanism. This is further supported by the NIEL/IEL ratios shown in Figure 3d. The results demonstrate that the radiation damage in the InAlGaN/GaN-based HEMT comprises both ionization and displacement damage, with ionization effects predominating. Figure 3d also shows that with increasing fluence, the proportion of NIEL increases initially and then levels off, with the 150 and 50 nm samples exhibiting higher NIEL proportions at higher fluences compared to the 100 nm sample. In summary, while displacement damage is significant in the proton-irradiated InAlGaN/GaN-based HEMT, the IEL component surpasses NIEL by an order of magnitude, indicating that IEL is the primary contributor to device performance degradation.

Figure 4 presents the drain current (*I*_d_) and transconductance (*g*_m_) characteristics as functions of gate voltage (*V*_g_) for each sample, both before and after proton irradiation. All postirradiation measurements were performed 4 days after irradiation to ensure radiation levels were below safe limits for personnel handling. Data shown are for a drain voltage (*V*_d_) of 10 V. The pristine *I*_d_ of the 100 nm sample exhibits the highest value among the three thicknesses, consistent with the Hall measurement results presented in the previous section. This superior performance of the 100 nm sample in the pristine state further supports the existence of an optimal channel thickness for maximizing 2DEG density and minimizing scattering effects; thinner channels suffer from reduced polarization-induced charge and increased interface scattering, while thicker channels may introduce more crystal defects that contribute to scattering. Similarly, the 100 nm sample also exhibits the highest peak transconductance (*g*_m_), directly reflecting the higher mobility observed in the Hall measurements.

**Figure 4:**
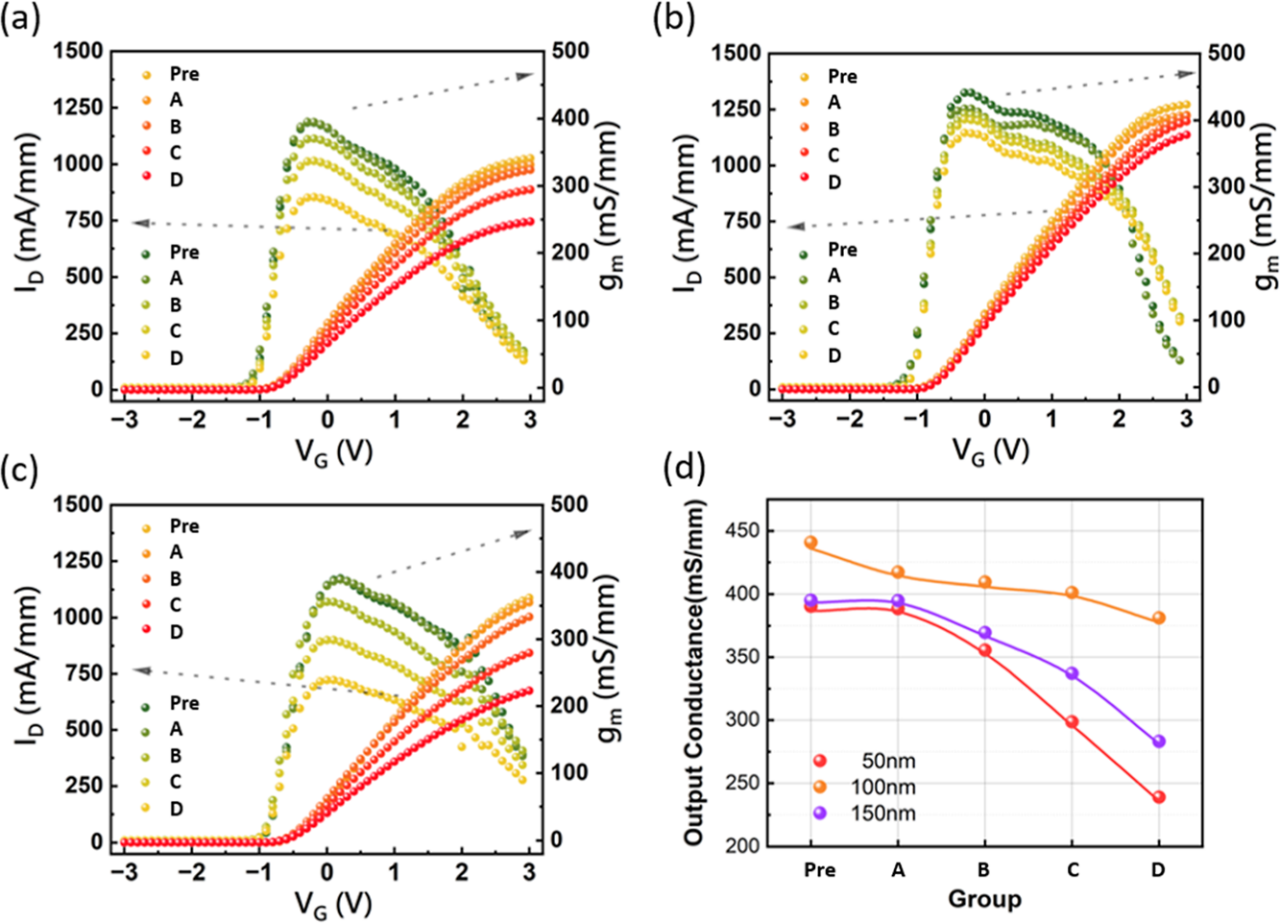
*I*_d_ and *g*_m_ as functions of *V*_g_ before and after proton irradiation for samples with channel thicknesses of (a) 150 nm, (b) 100 nm, and (c) 50 nm. The meaning of Pre, A, B, C, and D shown in this figure is the same as described in Figure 2. (d) Comparison of the peak transconductance (*g*_m,peak_) degradation for each thickness across various irradiation fluences.

Following irradiation with varying fluences, the 100 nm sample demonstrates the slowest degradation rate among the three thicknesses. This slower degradation rate in the 100 nm sample can be attributed to a combination of factors. First, the higher initial carrier concentration in the 100 nm sample provides a larger carrier pool to compensate for the reduction in mobility caused by radiation-induced defects. Second, the optimal balance between polarization-induced charge and scattering mechanisms in the 100 nm sample likely results in a less severe impact of radiation-induced defects. The increased defect density due to proton irradiation acts as scattering centers reducing mobility and *g*_m_. However, this effect is mitigated in the 100 nm sample because of the higher initial carrier concentration and favorable balance of scattering mechanisms. Thinner samples (50 nm) show a more rapid degradation because of the initial lower carrier concentration and significant interface scattering, making them more sensitive to the additional scattering caused by radiation defects. Conversely, thicker samples (150 nm) may suffer greater degradation due to a higher density of pre-existing defects, which are exacerbated by the additional radiation-induced defects. Figure 4d quantifies the degradation in peak *g*_m_ for each thickness at different fluences, clearly showing that the 100 nm sample exhibits less severe degradation than those with other thicknesses, reinforcing the conclusion that an optimal channel thickness exists for maximizing radiation hardness. The observed trends are consistent with the impact of radiation-induced defects on carrier scattering and the initial differences in carrier concentration and mobility across different channel thicknesses.

Figure 5 illustrates the variation in *I*_d_ as a function of drain-source voltage (*V*_ds_) for each channel thickness before and after proton irradiation. The data presented are for a gate voltage (*V*_g_) of 0 V. Consistent with previous observations, the pristine *I*_d_ of the 100 nm sample exhibits the highest value among the three thicknesses. Following proton irradiation with varying fluences, the 100 nm sample demonstrates the slowest degradation rate. Figure 5d shows the trend of contact resistance (*R*_c_) degradation for each thickness at different fluences, based on measurements from seven samples per thickness and fluence. The 150 nm sample exhibits the lowest pristine *R*_c_, consistent with its higher initial carrier concentration and potentially lower density of pre-existing defects. However, the 100 nm sample exhibits a less pronounced increase in *R*_c_ with increasing fluence than the 50 nm sample, while the 150 nm sample shows a significant increase in *R*_c_ at higher fluences. This behavior suggests that the 100 nm sample’s superior initial performance translates to improved resilience against radiation-induced increases in contact resistance. The observed trends in contact resistance further underscore the significance of optimizing channel thickness to minimize the impact of radiation-induced defects.

**Figure 5:**
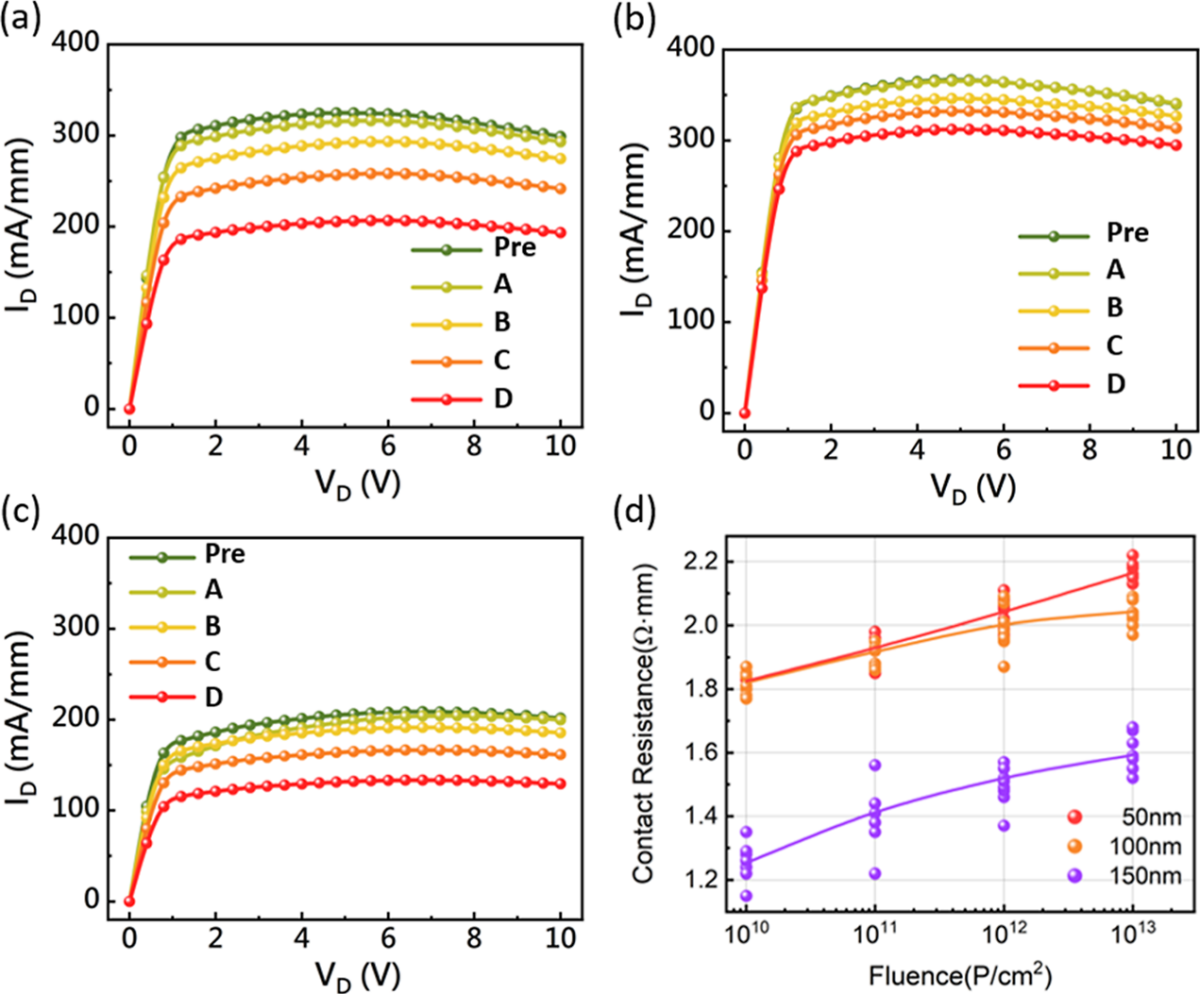
*I*_d_–*V*_d_ characteristics before and after proton irradiation treatment, for the channel thicknesses of (a) 150 nm (b) 100 nm and (c) 50 nm. The meaning of Pre, A, B, C, and D shown in this figure is the same as described in Figure 2. (d) Data by contact resistance for each thickness through different fluence.

An investigation into the effects of irradiation on gate characteristics was conducted via gate leakage current measurements, the results of which are presented in Figure 6. The relatively constant leakage current (approximately 10^–6^ to 10^–7^ mA/mm) observed across all channel thicknesses and irradiation fluences suggests a complex interplay of competing leakage mechanisms subtly affected by radiation-induced defects. A mechanistic analysis of postirradiation leakage is presented, differentiating between forward and reverse bias conditions and supported by the schematic diagrams in Figure 7.

**Figure 6:**
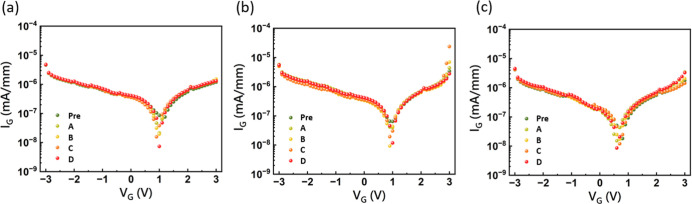
Forward and reverse leakage characteristics under various proton fluences for channel thicknesses of (a) 150 nm, (b) 100 nm, and (c) 50 nm. The meaning of Pre, A, B, C, and D shown in this figure is the same as described in Figure 2. The data presented are for a drain voltage (*V*_d_) of 0 V.

**Figure 7:**
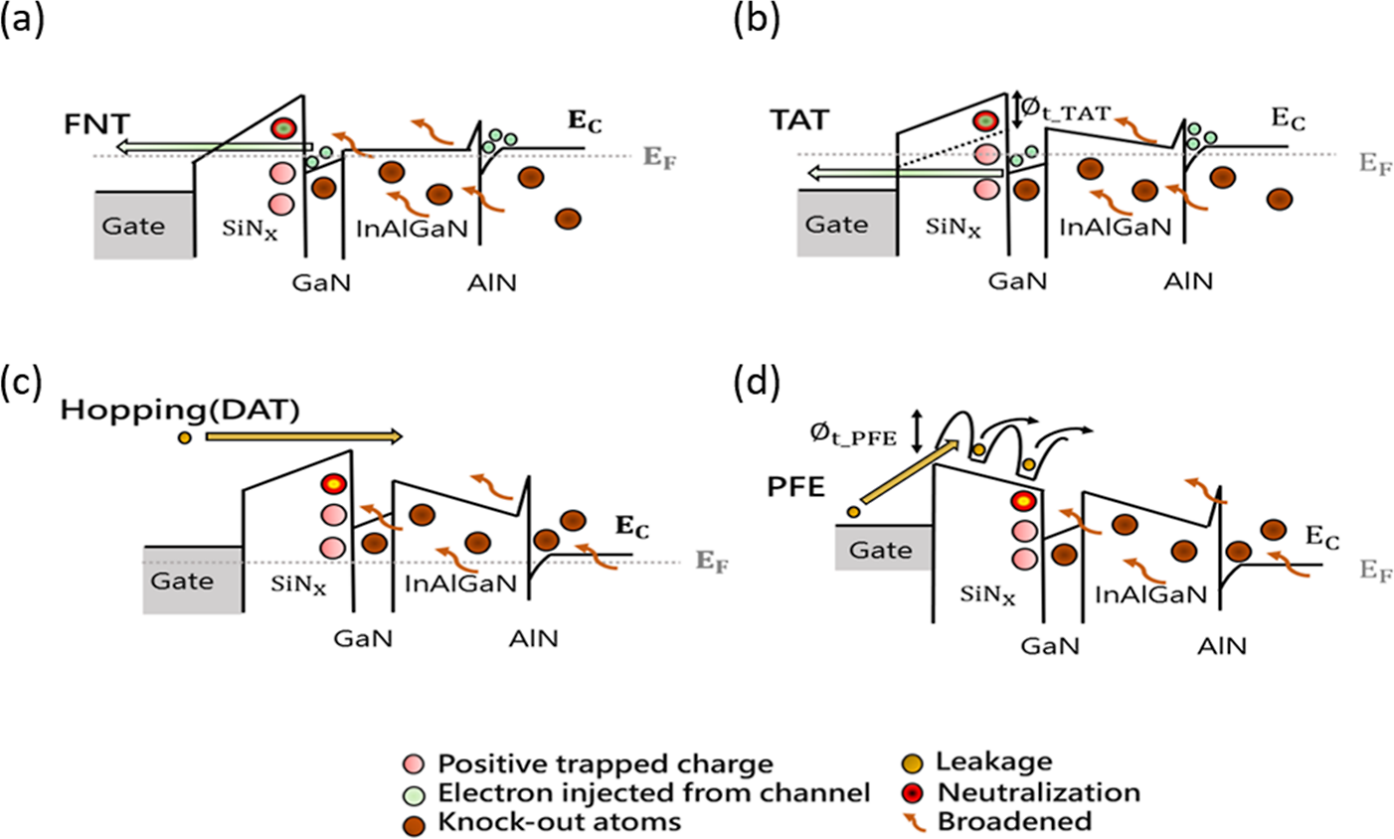
Schematic conduction band edge diagrams depicting the dominant charge transport mechanisms and the neutralization processes involving knock-on atoms generated by proton irradiation in different bias regions. These diagrams illustrate (a) Fowler–Nordheim tunneling (FNT), (b) trap-assisted tunneling (TAT), (c) hopping (defect-assisted tunneling, DAT), and (d) Poole–Frenkel emission (PFE).

In the forward bias region, two primary leakage mechanisms are generally considered: Fowler–Nordheim tunneling (FNT) and trap-assisted tunneling (TAT). FNT is characterized by a strong electric field dependence and a weak temperature dependence of the leakage current, enabling electron transport through the passivation layer and subsequent leakage from the GaN cap layer, as illustrated in Figure 7a. The observed reduction in FNT leakage current is attributed to an increase in the effective barrier height of the passivation layer. This suggests a mechanism involving the neutralization of positive traps and the generation of negative traps through electron capture from the GaN cap layer (or the 2DEG). Conversely, proton irradiation generates knock-on atoms, particularly a high concentration of knock-on atoms in the GaN channel layer (as shown by the high DPA values in Figure 3b). Upon relaxation, these atoms partially occupy defect sites within the passivation layer, directly impacting trap capture characteristics and partially counteracting the neutralization effect from electron capture, resulting in a relatively small change in FNT leakage current. Figure 7b shows the conduction band edge diagram relevant to trap-assisted tunneling (TAT). Separate electric field calculations were performed for the passivation and GaN cap layers, incorporating the effects of polarization charges at the III-nitride heterointerfaces/surface and interfacial charges at the passivation/GaN cap layer interface. The observation that the electric field across the passivation layer significantly exceeds that across the GaN cap layer supports the assertion that two-step TAT is the dominant leakage mechanism in this region. ϕt, TAT represents the trap energy level associated with TAT conduction. Proton irradiation generates knock-on atoms; upon relaxation, some of these atoms replace previously neutralized positive traps involved in two-step TAT. The incorporation of knock-on atoms into AlN/GaN interface traps within the passivation layer enhances electron mobility, extending into the GaN cap layer and counteracting the reduction in leakage current. This leads to a relatively constant gate leakage current under forward bias conditions.

In the reverse bias region, two dominant leakage mechanisms are typically considered: defect-assisted tunneling (DAT) and Poole–Frenkel emission (PFE). As illustrated in Figure 7c, DAT is characterized by weak, opposing electric fields across the passivation and GaN cap layers. Consequently, transport is primarily governed by the potential difference, and the resulting current density profile resembles thermionic emission.^40^ The magnitude of hopping DAT current is significantly influenced by the density of defects within the gate dielectric. Proton irradiation generates knock-on atoms which, upon relaxation, partially neutralize the hopping leakage current. Unlike the forward bias condition, the electron population in the GaN cap layer and at the AlN/GaN heterointerface does not exhibit significant leakage. Instead, under the influence of the reverse bias field, knock-on atoms distribute across various interfaces. Due to these competing effects, the leakage current stemming from the hopping (DAT) mechanism remains relatively constant. The observed dependence of leakage current on both electric field and temperature in the reverse bias region suggests the presence of Poole–Frenkel emission (PFE) current.^41^ This is further supported by the high electric field across the passivation layer under reverse bias conditions. Figure 7d illustrates the conduction band diagram and the PFE mechanism via a trap energy level, φt. Electrons initially trapped in localized states within the passivation layer may overcome the trap energy barrier through thermal excitation to transition into the conduction band. However, the neutralization of positive trapped charges via PFE is countered by the presence of relaxed knock-on atoms generated by proton irradiation, effectively maintaining the overall leakage path. Consequently, the competing effects result in a relatively constant gate leakage current in the reverse bias region.

A Monte Carlo simulation was employed to determine the defect distribution within the structure following irradiation with protons of varying energies (1–90 MeV). The simulation utilized a displacement threshold energy (TDE) of 20 eV for Ga atoms in GaN, consistent with previous reports.^42^ A fluence of 10^13^ protons was used for each energy to ensure statistically significant results. Interatomic interactions were treated as stochastic events, with probabilities determined by their respective cross sections. A random sampling method was employed to determine the occurrence of each interaction.

Figure 8a–f present the simulated proton distributions within each layer of the material at various incident energies. The simulation clearly demonstrates an increase in penetration depth with increasing incident proton energy. This is evident from the progression shown in the figures: from partial penetration of the AlGaN/AlN SL buffer layer at 1 MeV (Figure 8a), to near-complete penetration of all layers, including the HR-Si substrate, at 30 MeV (Figure 8e). Furthermore, the results for 90 MeV proton irradiation (Figure 8f) exhibit complete penetration. White points in the simulation represent protons; other colors denote different atomic species (detailed in Figure 3).

**Figure 8:**
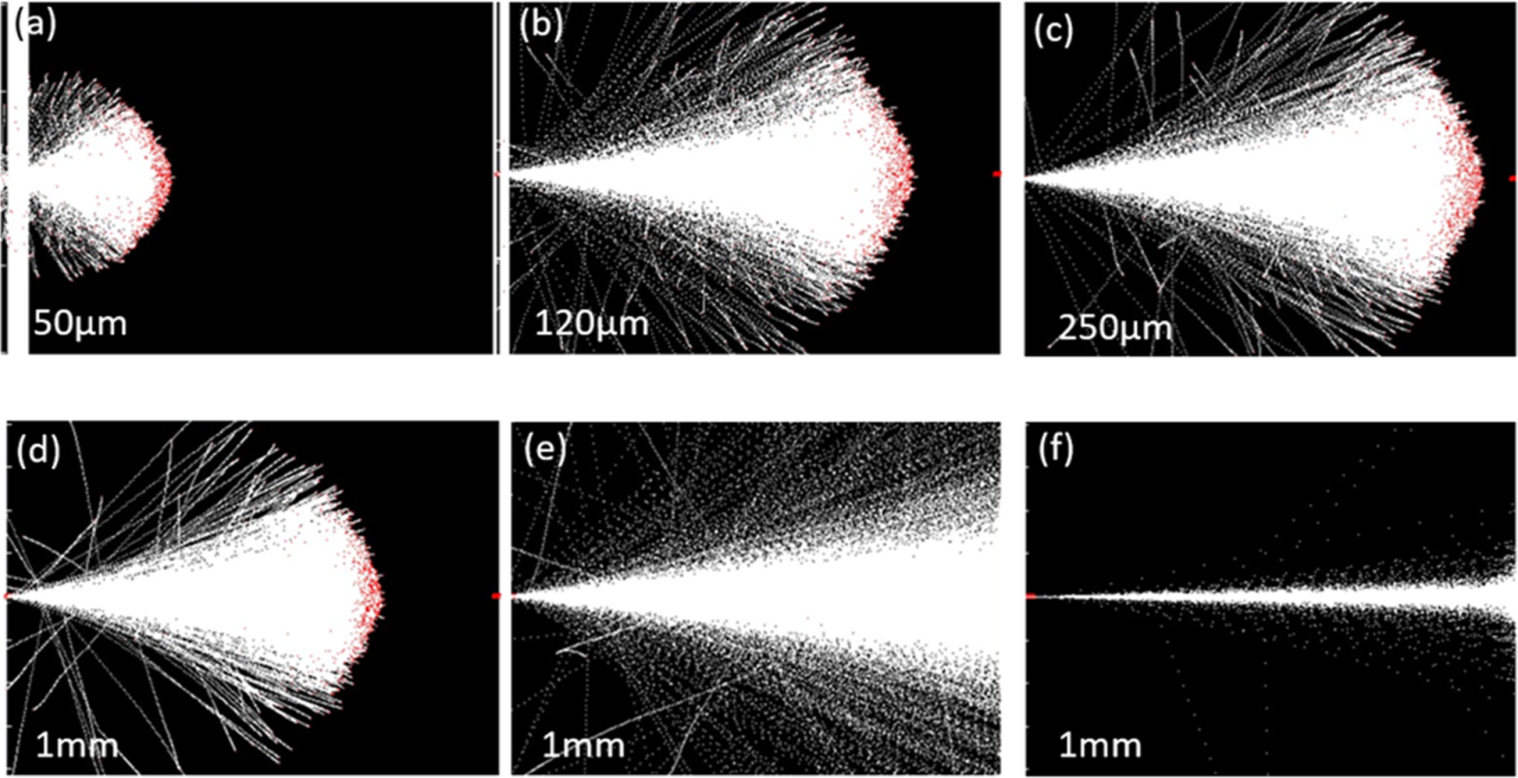
SRIM-simulated Monte Carlo distributions of particle impacts within the structure, penetrating from left to right, shown as cloud diagrams for incident proton energies of (a) 1 MeV (b) 3 MeV (c) 5 MeV (d) 10 MeV (e) 30 MeV and (f) 90 MeV. To clearly illustrate the particle distribution within the full structure, the scales of the target depth (the horizonal axis) in each subplot are individually adjusted and indicated in the lower-left corner of each respective figure.

A fraction of protons were deflected due to collisions and electrostatic interactions. Additionally, surface binding energies at material interfaces hindered recoil particle propagation, leading to a broadening of their spatial distribution. This broadening, analogous to the leakage path broadening discussed in Figure 7, is a consequence of the complex cascade of atomic displacements following the initial proton impact. Higher energy protons transfer greater energy upon impact, resulting in a more extensive cascade and a broader distribution of displaced atoms. The observed complete penetration at lower energies strongly suggests that 90 MeV protons will also penetrate the entire structure, leading to a uniformly distributed generation of defects across all layers. These simulation results provide a framework for understanding the type and distribution of defects generated by 90 MeV irradiation. Furthermore, this uniform defect distribution is expected to result in a more homogeneous impact on device performance compared to the nonuniform defect distribution observed at lower energies.

S-parameter measurements were performed up to 67 GHz, sweeping *V*_gs_ from −3 to 3 V while maintaining *V*_ds_ at 10 V. Small-signal models were extracted at each bias point. The cutoff frequency (*f*_*t*_) and maximum oscillation frequency (*f*_max_) were subsequently determined from the small-signal current gain (*H*_21_) and unilateral power gain (*U*). For radiation experiments, devices of identical channel thickness were divided into five groups (ten samples per group) representing different proton fluences: pristine (blue), and irradiated at 2 × 10^10^, 2 × 10^11^, 2 × 10^12^, and 2 × 10^13^ cm^–2^ (red). The measured *f*_*t*_ and *f*_max_ are shown in Figure 9a,b, respectively. The pristine 100 nm sample exhibited the highest measured values of *f*_*t*_ (58.4 GHz) and *f*_max_ (122.7 GHz), representing the peak values recorded for each parameter. For all channel thicknesses, both *f*_*t*_ and *f*_max_ decreased with increasing proton fluence. Notably, the 100 nm sample showed the least degradation in both parameters (*f*_*t*_ = 55.2 GHz and *f*_max_ = 94.4 GHz after 2 × 10^13^ cm^–2^ irradiation), confirming the superior electrical performance of the 100 nm channel thickness under 90 MeV proton irradiation, as observed previously in DC measurements. This superior performance is evident both before and after irradiation.

**Figure 9:**
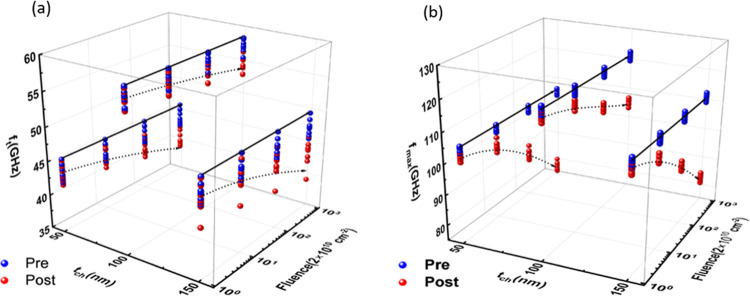
Three-dimensional representations of the degradation in (a) cutoff frequency (*f*_*t*_) and (b) maximum oscillation frequency (*f*_max_) as functions of channel thickness (*t*_ch_) and proton fluence. The distribution of blue points represents the measurement values obtained prior to irradiation, while the red points denote the values measured after irradiation at varying flux levels.

## Conclusions

InAlGaN has emerged as a promising alternative to AlGaN and InAlN, offering improved polarization and charge density, but deep-level traps in the buffer layer can hinder performance. This study examines the radiation response of InAlGaN HEMTs with varying channel thicknesses under proton irradiation, relevant to LEO environments. Results show that a 100 nm channel offers superior radiation hardness, maintaining higher mobility, lower sheet resistance, and stable DC and RF performance compared to 50 and 150 nm channels. The 100 nm thickness optimizes the balance between performance and radiation resistance, making it ideal for LEO applications. This research provides insights into the design of radiation-resistant GaN-based electronics for future space missions. This work could be extended by exploring other material combinations and device structures, including alternative buffer layers or advanced passivation techniques. It could guide future research on radiation-hardened electronics for deep-space missions, where radiation exposure is more extreme.

## Supplementary Material



## Notes

The Supporting Information provides details of arrangement of experimental setup and preirradiation Hall measurements from epitaxial layers of varying thicknesses (PDF)
