# Supplementary material for: Impact of Channel Effects on Radiation-Hardened InAlGaN HEMTs for Low-Earth-Orbit Applications
Source: ACS Omega. 2025 May 21;10(21):22247–56. doi: 10.1021/acsomega.5c02838 (PMC12138609; doi:10.1021/acsomega.5c02838)
Supplement: Supplementary file 1 [file ao5c02838_si_001.pdf]

Supporting Information

Impact of Channel Effects on Radiation-Hardened InAlGaN HEMTs for Low-Earth-Orbit Applications

Shao-Kuan Lee[1], *You-Chen Weng*[2], *Chien-Yuan Huang*[1], *Edward-Yi Chang*[1,3], and *Yuan-Chieh Tseng*[1,3]*

[1]International College of Semiconductor Technology, National Yang Ming Chiao Tung University, 30010, Hsinchu, Taiwan

[2]College of Photonics, National Yang Ming Chiao Tung University, 71150, Tainan,Taiwan

[3]Department of Materials Science and Engineering, National Yang Ming Chiao Tung University, 30010, Hsinchu, Taiwan

*yctseng1978@nycu.edu.tw

Figure S1 The arrangement of experimental setup

Figure S1. depicts the experimental irradiation setup. Irradiation was conducted at the Radiation Research Core Laboratory, Chang Gung Memorial Hospital, Taiwan, utilizing a 90 MeV proton beam from a cyclotron. Beam selection was achieved via a switching magnet. Subsequent beam focusing, necessitated by inherent Coulombic repulsion, was accomplished using a pair of quadrupole magnets, precisely directing the beam to the irradiation platform. Spatial beam uniformity was ensured by a double-scattering system, while penetration depth was controlled using a range modulation wheel (RMW). This RMW facilitates the generation of a spread-out Bragg peak (SOBP) through the superposition of dose contributions from protons of varying energies, resulting in a uniform dose plateau. Precise irradiation was achieved by manipulating the SOBP width. Finally, a collimator defined the irradiation field dimensions.

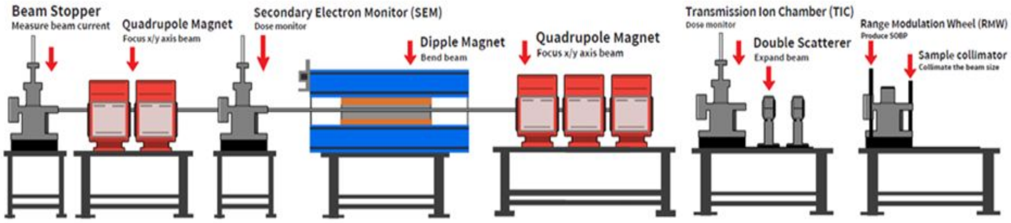

Figure S1. Schematic illustrating the key components of the experimental setup used for proton irradiation. The radiation source extends from the left side to the right side of the figure, and the object to be irradiated is fixed at a position 100 centimeters away from the Sample collimator on the far right for the radiation experiment.

Table S1 Pre-irradiation characterization of the epitaxial layers

Pre-irradiation measurements, averaged across ten samples per structure, revealed a strong correlation between GaN channel thickness and material properties. Specifically, the 100 nm GaN channel exhibited the highest electron mobility (μ=1940 $cm^2/V\cdot s$), a consequence of the highest 2DEG density arising from polarization effects. This high 2DEG density is a result of the optimal balance between polarization-induced charge and scattering mechanisms[31,32,34]; thinner channels (50 nm) exhibit lower 2DEG density due to reduced polarization effects and increased interface scattering, resulting in lower mobility[31,32,34]. Conversely, thicker channels (150 nm), while showing increased sheet carrier concentration, do not exhibit a proportionally higher mobility due to limitations in polarization-induced charge and increased scattering from crystal defects which are more prevalent in thicker layers[31,32,34]. A monotonic increase in sheet carrier concentration was observed with increasing GaN thickness, resulting in a correspondingly inverse relationship with sheet resistance (Rsh), ranging from approximately 353.2 Ω/sq (50 nm) to 199.7 Ω/sq (150 nm).

Table S1. A comparison of sheet resistance, 2DEG mobility (μ), and carrier concentration for 50 nm, 100 nm, and 150 nm channel thicknesses.

Channel thickness(nm)	R_{sh}(Ω/sq)	μ(cm^2/V-s)	n_s($10^{13}/cm^2$)
50	353.2	1780	0.99
100	220.7	1940	1.45
150	199.7	1880	1.66
